# 122. Impact of Infectious Diseases Consultation in Patients with Candidemia at a Large Multi-site Healthcare System Providing Telemedicine Services

**DOI:** 10.1093/ofid/ofab466.122

**Published:** 2021-12-04

**Authors:** Katie Hammer, Andrew Shifflet, Megan Petteys, Rohit Soman, Julie E Williamson, Leigh Ann Medaris, zainab shahid

**Affiliations:** 1 Atrium Health, SC, SC; 2 Levine cancer Institute, charlotte, North Carolina

## Abstract

**Background:**

*Candida* species are the most common cause of fungemia and are associated with high mortality. Management concordant with the Infectious Diseases Society of America guidelines and infectious diseases consultation (IDC) have been shown to lower mortality in patients with candidemia. The purpose of this study was to compare in-hospital mortality at a large multi-site healthcare system, including sites providing IDC via telemedicine services, in patients with candidemia with and without IDC.

**Methods:**

This was a retrospective, observational cohort study completed at ten sites of Legacy Atrium Health in Charlotte Metro, NC, USA; at five sites, IDC is performed via telemedicine. Adult hospitalized patients identified with candidemia were enrolled May 2018-June 2019. The primary outcome was in-hospital mortality of IDC and non-IDC patients. Secondary outcomes included obtainment of repeat blood cultures, receipt of antifungal treatment, duration of therapy, removal of central venous lines (CVC) when present, and ophthalmological examination. Fisher’s exact, Chi-Square, or two-tailed Student’s t-test were used for demographics, primary and secondary outcomes as appropriate.

**Results:**

A total of 126 patients were enrolled: 103 (82%) in the IDC group and 23 (18%) in the non-IDC group (Table 1). Mortality was significantly lower, and rates of repeat blood culture obtainment and receipt of antifungal treatment were significantly higher in patients with IDC (Table 2). Other outcomes including duration of therapy, removal of CVC, repeat cultures within 48 hours, and ophthalmological examination were not statistically different between groups.

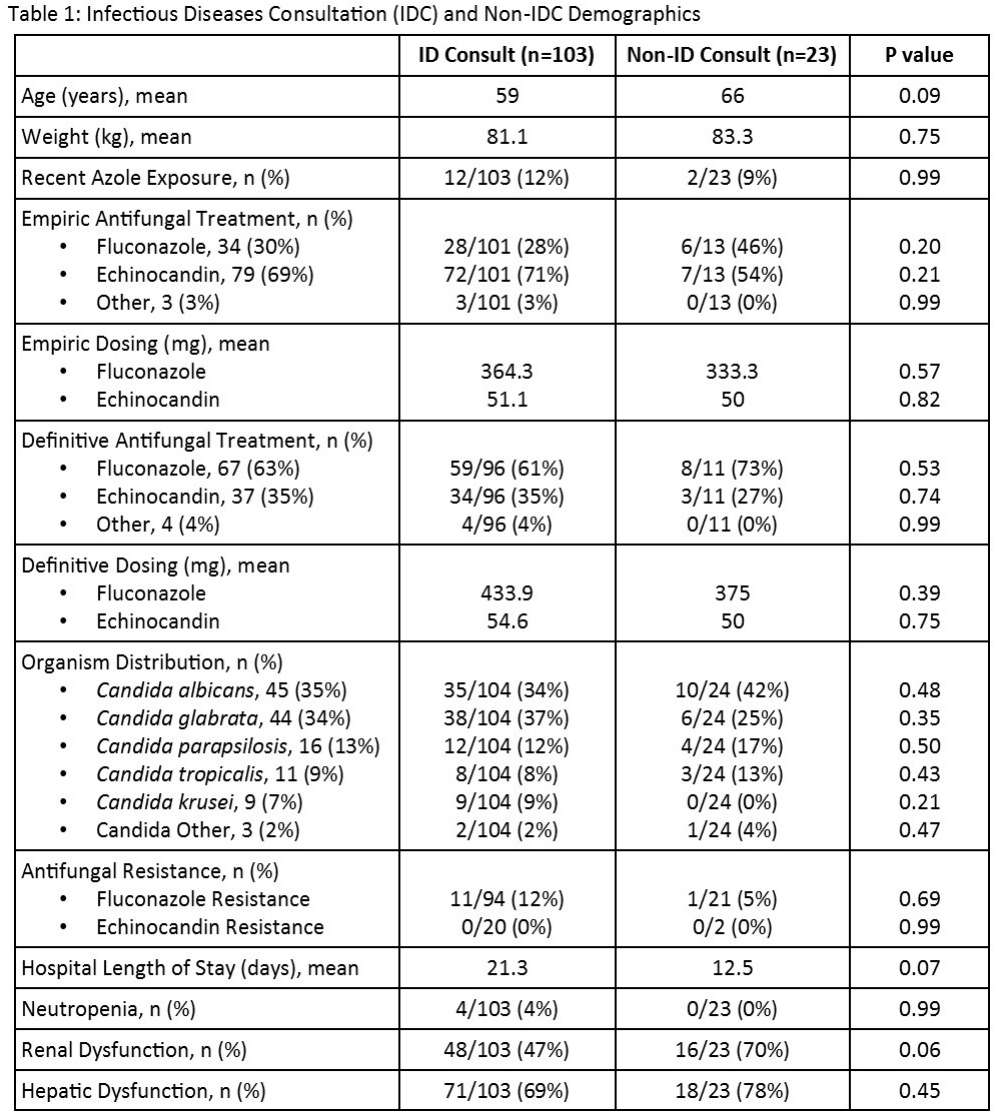

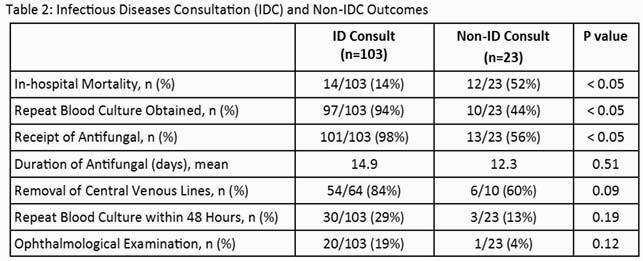

**Conclusion:**

This study is the first multi-site healthcare system providing telemedicine services to evaluate the impact of IDC on candidemia mortality. Ophthalmological examination rates were low in both groups, highlighting a potential area for improvement. IDC had significantly lower mortality, higher rates of antifungal treatment, and higher rates of repeat blood culture obtainment. IDC should be strongly considered in all patients with candidemia.

**Disclosures:**

**All Authors**: No reported disclosures

